# Systemic, asymptomatic skeletal muscle infiltration by MALT lymphoma: Defining a 'silent infiltrator' phenotype with 18F-FDG PET/CT

**DOI:** 10.1016/j.lrr.2026.100573

**Published:** 2026-02-20

**Authors:** Yasuyuki Takahashi, Ken Naganuma, Yuka Tanaka, Noriyuki Sakata, Taisuke Kawada, Masahiro Kizaki, Shuji Momose, Morihiro Higashi, Takayuki Tabayashi

**Affiliations:** a Department of Hematology, Saitama Medical University, Saitama Medical Center, Kawagoe, Saitama, Japan; b Department of Hematology, Otemae Hospital, Osaka, Japan; c Department of Pathology, Saitama Medical University, Saitama Medical Center, Kawagoe, Saitama, Japan

**Keywords:** Malt lymphoma, Skeletal muscle, ¹⁸F-FDG PET/CT, Asymptomatic, Extranodal Lymphoma

## Abstract

We report a rare phenotype of Stage IV extranodal marginal zone lymphoma (MALT lymphoma) manifesting as diffuse, asymptomatic skeletal muscle and bone marrow infiltration in a 60-year-old female. Despite a high tumor burden and intense ¹⁸F-FDG avidity (SUVmax 8.8), muscle enzymes remained normal, indicating a non-destructive "silent infiltrator" growth pattern. The diagnosis was confirmed via biopsy (CD20+, CD5-, CyclinD1-) and negative MYD88 L265P mutation status, excluding lymphoplasmacytic lymphoma. The patient achieved a Complete Metabolic Response following Bendamustine-Rituximab therapy. This case underscores the utility of PET/CT in detecting occult systemic disease and defines a unique, indolent clinical variant of muscular MALT lymphoma.

## 1 Introduction

Extranodal marginal zone lymphoma of mucosa-associated lymphoid tissue (MALT lymphoma) is an indolent B-cell neoplasm that arises in extranodal tissues, typically as a result of chronic inflammation. While the stomach is the most common site, non-gastric presentations occur in the ocular adnexa, lung, and salivary glands. Skeletal muscle involvement is a rare manifestation, accounting for <1 % of all non-Hodgkin lymphomas and is predominantly associated with Diffuse Large B-Cell Lymphoma (DLBCL) or disseminated Follicular Lymphoma [1]. When MALT lymphoma does involve muscle, reported cases invariably describe localized, painful masses mimicking sarcoma or myositis.

Herein, we report an unprecedented case of diffuse, systemic skeletal muscle and bone marrow infiltration by MALT lymphoma that remained clinically asymptomatic ("silent") and biochemically occult. This case defines a novel clinicopathologic phenotype and validates the utility of 18F-FDG PET/CT and MYD88 mutation analysis in correctly classifying and managing atypical extranodal presentations.

## 2 Case presentation

**Clinical History and Physical Examination:**

A 60-year-old female presented with a painless swelling on her forehead, which she had noted for approximately 10 years. She denied any B symptoms (fever, night sweats, weight loss), bone pain, myalgia, or muscle weakness. Her ECOG performance status was 0.

Physical examination revealed a firm, non-tender mass over the left frontalis muscle. Critically, systemic palpation identified firm, non-tender masses within both biceps’ muscles, which were synchronous findings not previously noted by the patient. There was no palpable lymphadenopathy in the cervical, supraclavicular, axillary, or inguinal regions. Hepatosplenomegaly was absent. The patient had no history of occupational exposure to agricultural chemicals or industrial solvents.

**Laboratory Findings:**

Complete blood count was normal. Serum lactate dehydrogenase (LDH) and C-reactive protein (CRP) were within normal limits. Markers of muscle injury were strictly normal: Creatine Kinase (CK) was 37 U/L (reference range 45–163 U/L), and Aldolase was 3.1 U/L (reference range <7.6 U/L). Conversely, Soluble IL-2 receptor (sIL-2R) was markedly elevated at 1170 U/mL (reference range 145–519 U/L), suggesting a high tumor burden [2].

To rule out Waldenström Macroglobulinemia (WM) and multiple myeloma, serum protein electrophoresis (SPEP) and immunofixation were performed, revealing no monoclonal gammopathy. Beta-2 microglobulin was within normal limits (Table 1).

**Table 1:** Summary of laboratory, pathological, and imaging findings at initial presentation.

Category	Parameter	Value	Reference Range
Hematology	White Blood Cell Count	5800/μL	4000–10,000/μL
	Hemoglobin	12.3 g/dL	12.0–16.0 g/dL
	Platelet Count	199,000/μL	150,000–450,000/μL
Biochemistry	Lactate Dehydrogenase (LDH)	190 U/L	124–222 U/L
	Creatine Kinase (CK)	37 U/L	45–163 U/L
	Aldolase	3.1 U/L	<7.6 U/L
	Soluble IL-2 Receptor	1170 U/mL	145–519 U/mL
Pathology	Lineage (IHC, Muscle)	CD20+, CD3-	B-cell origin
	Immunophenotype (IHC, Muscle)	CD5-, CD10-, Cyclin D1-	Excludes MCL, CLL/SLL, FL
	Translocation (FISH, Muscle)	API2-MALT1 t(11;18) negative	Absence of common translocation
	Mutation	MYD88 L265P mutation negative	Excludes WM/LPL
Imaging	18F-FDG PET/CT	Diffuse, intense uptake in systemic skeletal muscle and bone marrow	Pathological uptake

**Imaging:**

**Fig. 1:**
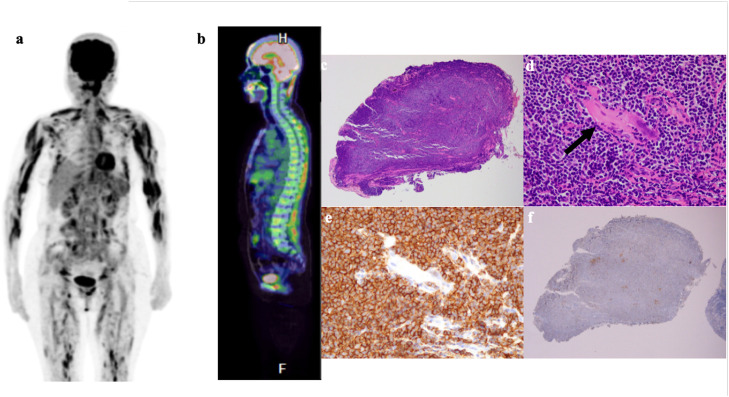
Radiological and histopathological features of MALT lymphoma with systemic skeletal muscle infiltration. (a) ¹⁸F-FDG PET/CT maximum intensity projection (MIP) image shows markedly increased, diffuse FDG uptake throughout the skeletal muscles of the trunk and extremities, as well as in the bone marrow. (b) ¹⁸F-FDG PET/CT sagittal fusion image demonstrates diffuse hypermetabolism in the vertebral bone marrow and paraspinal muscles. (c) Hematoxylin and eosin (H&E) stain of the frontalis muscle biopsy (low-power view) shows effacement of the normal muscle architecture by a dense, diffuse infiltrate of small lymphoid cells. (d) H&E stain (high-power view) reveals a monotonous proliferation of small to medium-sized lymphocytes with irregular nuclear contours (centrocyte-like cells) infiltrating between atrophic skeletal muscle fibers (arrow). (e) Immunohistochemical staining for CD20 shows diffuse and distinct membrane positivity in tumor cells, confirming their B-cell origin. (f) Immunohistochemical staining for CD3 is negative in tumor cells, with only scattered reactive T-cells in the background.

Whole-body 18F-FDG PET/CT revealed diffuse, intense FDG uptake throughout the skeletal muscles of the trunk, upper and lower extremities, and the frontalis muscle. The SUVmax of the muscular lesions reached 8.8. Diffuse hypermetabolism was also noted in the axial skeleton bone marrow. No pathologically enlarged lymph nodes were identified (Fig. 1, Table 1).

**Pathology:**

Incisional biopsy of the frontalis muscle demonstrated a dense infiltrate of small- to medium-sized lymphocytes with irregular nuclei (centrocyte-like cells) insinuating between skeletal muscle fibers without frank necrosis or destruction of the myofibers. Immunohistochemistry (IHC) showed the neoplastic cells were CD20+, Bcl-2+, and negative for CD3, CD5, CD10, CD23, and Cyclin D1. Kappa light chain restriction was demonstrated by in situ hybridization.

A bone marrow biopsy confirmed concordant infiltration by small B-lymphocytes with an identical immunophenotype and Kappa restriction. Plasmacytic differentiation was not prominent.

To definitively exclude Lymphoplasmacytic Lymphoma (LPL), MYD88 L265P mutation analysis was performed on the biopsy specimen and was negative [3]. FISH analysis was negative for the t (11;18) (q21; q21) translocation (Fig. 1, Table 1).

**Diagnosis and Staging:**

An upper gastrointestinal endoscopy (EGD) was performed to screen for a primary gastric lesion; macroscopic findings were normal, and biopsies were negative for lymphoma and *Helicobacter pylori*. Based on the systemic involvement of skeletal muscles and bone marrow, the patient was diagnosed with Stage IV Extranodal Marginal Zone Lymphoma (MALT type) according to the Lugano classification [4].

**Treatment and Outcome:**

Given the high systemic tumor burden and Stage IV disease, systemic chemoimmunotherapy was indicated. The patient was treated with **Bendamustine** (90 mg/m2, days 1–2) and **Rituximab** (375 mg/m2, day 1) (BR regimen) every 28 days. The BR regimen was selected ("aptly" chosen) for its favorable toxicity profile, specifically the lack of alopecia and neurotoxicity, to preserve the quality of life in this asymptomatic patient [5].

After 4 cycles, a restaging PET/CT showed complete resolution of all abnormal FDG uptake, confirming a Complete Metabolic Response (CMR) [6]. A repeat bone marrow biopsy was performed, which confirmed histologic clearance (Complete Remission). The patient remains in sustained remission 3 years post-therapy.

## 3 Discussion

This case illustrates a unique "Silent Infiltrator" phenotype of MALT lymphoma, characterized by a profound disconnect between massive, systemic tumor burden and the absence of clinical symptoms or tissue destruction.

**The "Silent Infiltrator" Phenotype:**

Unlike aggressive lymphomas (e.g., DLBCL) or sarcomas that destructively invade muscle causing pain and enzyme elevation, the MALT lymphoma cells in this case exhibited a "non-destructive" interstitial growth pattern [7]. They permeated the fascial and inter-muscular planes, preserving the structural integrity of the myocytes, as evidenced by normal CK and Aldolase levels. This behavior aligns with the tendency of MALT lymphoma to colonize existing tissue architecture (e.g., lymphoepithelial lesions) rather than efface it [8].

**Diagnostic Precision: Excluding LPL:**

The combination of bone marrow and soft tissue involvement strongly mimics Lymphoplasmacytic Lymphoma (LPL). Differentiating MALT from LPL is critical due to divergent treatment implications (e.g., BTK inhibitors for WM). In this case, the absence of an IgM paraprotein and the negative MYD88 L265P mutation status were decisive. MYD88 L265P is present in >90 % of LPL cases but is rare (<10 %) in MALT lymphoma, making it a robust exclusionary biomarker in this diagnostic dilemma [9,10].

**Revisiting Staging Paradigms:**

While MALT lymphoma has historically been considered to have variable FDG avidity, this case supports recent data suggesting high sensitivity of PET/CT for extranodal variants [4,11]. The SUVmax of 8.8 typically raises concern for high-grade transformation; however, this case proves that high SUV in MALT can reflect high tumor cell density rather than aggressive grade [11]. Without PET/CT, the occult bone marrow and systemic muscle involvement would likely have been missed (given the normal physical exam and blood counts), potentially leading to a misdiagnosis of localized (Stage I) disease and inappropriate local therapy. Although bone marrow biopsy alone would define Stage IV, the PET/CT provided the rationale to suspect systemic disease and guided the confirmatory biopsies.

**Therapeutic Rationale:**

The choice of Bendamustine-Rituximab was optimal ("apt") for this scenario. While R-CHOP is a standard for aggressive lymphomas, its toxicity profile is suboptimal for an asymptomatic patient. The BR regimen has demonstrated non-inferiority to R-CHOP in indolent lymphomas with significantly less toxicity, successfully inducing a durable complete remission in this patient while maintaining her asymptomatic status [6,12].

## 4 Conclusion

We report the first documented case of diffuse, systemic, and asymptomatic skeletal muscle MALT lymphoma. This "silent infiltrator" phenotype underscores the necessity of comprehensive metabolic staging (PET/CT) and molecular profiling (MYD88) in atypical extranodal lymphomas. The complete response to Bendamustine-Rituximab validates this regimen as a highly effective and well-tolerated strategy for high-burden, indolent disease.

## Ethics approval and consent to participate

The patient provided written informed consent for the publication of this case report and any accompanying images. Institutional review board approval was not required for this single case report.

## Declaration of generative AI and AI-assisted technologies in the manuscript preparation process

During the preparation of this work, the author(s) used generative AI and AI-assisted technologies to improve language and readability. After using these tools, the author(s) reviewed and edited the content as needed and take full responsibility for the content of the publication.

## Funding

No funding was received for this study.

## Availability of data and materials

The data generated and/or analyzed during the current study are included in this published article.

## CRediT authorship contribution statement

**Yasuyuki Takahashi:** Writing – original draft, Data curation. **Ken Naganuma:** Data curation. **Yuka Tanaka:** Writing – original draft, Investigation, Data curation. **Noriyuki Sakata:** Data curation. **Taisuke Kawada:** Data curation. **Masahiro Kizaki:** Writing – review & editing. **Shuji Momose:** Writing – review & editing, Data curation. **Morihiro Higashi:** Writing – review & editing. **Takayuki Tabayashi:** Writing – review & editing.

## Declaration of competing interest

The authors declare that they have no competing interests.

## References

[1] Travis W.D., Banks P.M., Reiman H.M. (1987). Primary extranodal soft tissue lymphoma of the extremities. Am. J. Surg. Pathol..

[2] Yoshida N., Oda M., Kuroda Y. (2013). Clinical significance of sIL-2R levels in B-cell lymphomas. PLoS One.

[3] Treon S.P., Xu L., Yang G. (2012). MYD88 L265P somatic mutation in Waldenström's macroglobulinemia. N. Engl. J. Med..

[4] Cheson B.D., Fisher R.I., Barrington S.F. (2014). Recommendations for initial evaluation, staging, and response assessment of Hodgkin and non-hodgkin lymphoma: the Lugano classification. J. Clin. Oncol..

[5] Flinn I.W., van der Jagt R., Kahl B.S. (2014). Randomized trial of bendamustine-rituximab or R-CHOP/R-CVP in first-line treatment of indolent NHL or MCL: the BRIGHT study. Blood.

[6] Salar A., Domingo-Domenech E., Panizo C. (2017). Long-term results of a phase 2 study of rituximab and bendamustine for mucosa-associated lymphoid tissue lymphoma. Blood.

[7] Isaacson P.G., Du M.Q. (2004). MALT lymphoma: from morphology to molecules. Nat. Rev. Cancer.

[8] Isaacson P.G., Wotherspoon A.C., Diss T., Pan L.X. (1991). Follicular colonization in B-cell lymphoma of mucosa-associated lymphoid tissue. Am. J. Surg. Pathol..

[9] Martinez-Lopez A., Curiel-Olmo S., Mollejo M. (2015). MYD88 (L265P) somatic mutation in marginal zone B-cell lymphoma. Am. J. Surg. Pathol..

[10] Xu L., Hunter Z.R., Yang G. (2013). MYD88 L265P in Waldenström macroglobulinemia, immunoglobulin M monoclonal gammopathy, and other B-cell lymphoproliferative disorders using conventional and quantitative allele-specific polymerase chain reaction. Blood.

[11] Perry C., Herishanu Y., Metzer U. (2007). Diagnostic accuracy of PET/CT in patients with extranodal marginal zone MALT lymphoma. Eur. J. Haematol..

[12] Rummel M.J., Niederle N., Maschmeyer G. (2013). Bendamustine plus rituximab versus CHOP plus rituximab as first-line treatment for patients with indolent and mantle-cell lymphomas: an open-label, multicentre, randomised, phase 3 non-inferiority trial. Lancet.

